# The metagenomic next-generation sequencing in diagnosing central nervous system angiostrongyliasis: a case report

**DOI:** 10.1186/s12879-020-05410-y

**Published:** 2020-09-21

**Authors:** Li Feng, Aiwu Zhang, Jiali Que, Hongyan Zhou, Haiyan Wang, Yuanlin Guan, Cunzhou Shen, Xunsha Sun, Rong Lai, Fuhua Peng, Huiyu Feng, Ling Chen

**Affiliations:** 1 Department of Neurology, National Key Clinical Department and Key Discipline of Neurology, The First Affiliated Hospital, Sun Yat-sen University, Guangzhou, China; 2 Department of Intensive Care Unit, The Guangdong Second Provincial General Hospital, Guangzhou, China; 3 Department of Neurology, The Seventh Affiliated Hospital, Sun Yat-sen University, Shenzhen, China; 4 Hugobiotech Co. Ltd, Beijing, China; 5 Department of Neurology, The Third Affiliated Hospital, Sun Yat-sen University, Guangzhou, China

**Keywords:** Angiostrongyliasis, Eosinophilic meningoencephalitis, Metagenomic next-generation sequencing, Diagnosis, Case report

## Abstract

**Backgrounds:**

The incidence of angiostrongyliasis is increasing in recent decades due to the expanding endemic areas all over the world. Clinicians face tremendous challenge of diagnosing angiostrongyliasis because of the lack of awareness of the disease and less effective definitive laboratory tests.

**Case presentation:**

A 27-year-old man initially manifested skin itching, emesis, myalgia and quadriparesis. With progressive weakness of four limbs and elevated protein in the cerebrospinal fluid (CSF), he was diagnosed as Guillain-Barré syndrome and treated with intravenous methylprednisolone and immunoglobulin. However, the patient deteriorated with hyperpyrexia, headache and then persistent coma. The routine tests for *Angiostrongylus cantonensis (A. cantonensis)* with both the CSF and the serum were all negative. In contrast, the metagenomic next-generation sequencing (mNGS) was applied with the serum sample and the CSF sample in the middle phase. The central nervous system (CNS) angiostrongyliasis was diagnosed by mNGS with the mid-phase CSF, but not the mid-phase serum. At the same time, the CSF analysis revealed eosinophils ratio up to 67%. The discovery of *A. cantonensis* was confirmed by PCR with CSF later. Unfortunately, the patient died of severe angiostrongyliasis. During his hospitalization, mNGS was carried out repeatedly after definitive diagnosis and targeted treatment. The DNA strictly map reads number of *A. cantonensis* detected by mNGS was positively correlated with the CSF opening pressure and clinical manifestations.

**Conclusions:**

The case of *A. cantonensis* infection highlights the benefit of mNGS as a target-free identification in disclosing the rare CNS angiostrongyliasis in the unusual season, while solid evidence from routine clinical testing was absent. The appropriate sample of mNGS should be chosen according to the life cycle of *A. cantonensis*. Besides, given the fact that the DNA reads number of *A. cantonensis* fluctuated with CSF opening pressure and clinical manifestations, whether mNGS could be applied as a marker of effectiveness of treatment is worth further exploration.

## Background

*Angiostrongylus cantonensis* (*A. cantonensis*) is a zoonotic pathogen, which occasionally causes human angiostrongyliasis with the main clinical manifestation as eosinophilic meningitis [1]. Although the parasite is usually endemic to Southeast Asia and the Pacific Basin, the global distribution of *A. cantonensis* is expanding, and the incidence of angiostrongyliasis among humans is increasing, too [2]. Angiostrongyliasis usually presents as eosinophilic meningitis or meningoencephalitis with protean clinical manifestations including paresthesia, headache, weakness, nerve pain, coma or even death [2]. The patients with angiostrongyliasis who received appropriate treatment in the early phase usually have a favorable prognosis [3]. However, approximately 10% of the untreated or delayed treated patients may develop severe central nervous system (CNS) angiostrongyliasis with mortality as high as 79–91% [4–6]. Moreover, the treatment of angiostrongyliasis beyond the early phase is still controversial, especially for severe CNS angiostrongyliasis [7]. Herein, achieving the definitive diagnosis of angiostrongyliasis as early as possible is essential for patients to survive. Unfortunately, neither the eosinophilic peak nor the positive antibody against *A. cantonensis* would appear in the early phase of angiostrongyliasis [8], not mentioning the insufficient awareness of angiostrongyliasis among doctors beyond the traditional endemic areas. The diagnosis of angiostrongyliasis has been a great challenge for clinicians. The metagenomic next-generation sequencing (mNGS), which takes the advantage of allowing target-independent identification of all microbes in a sample, has been more and more applied in the diagnosis of infectious diseases [9]. In this case, we used mNGS to identify the parasitic etiology in an unusual case in which the common laboratory testing of the serum and the cerebrospinal fluid (CSF) were all negative. Moreover, the case also provided new prospect for the first time that the sensitivity of mNGS with CSF sample might be higher than that with serum sample for angiostrongyliasis in the middle phase. The mNGS was also applied to monitor the progress and treatment effectiveness of the case.

## Case presentation

**Fig. 1:**
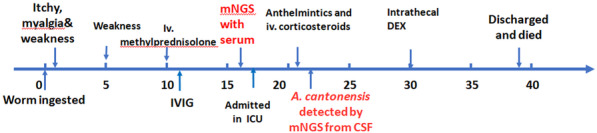
The time line of the case’ medical process. Dex: dexamethasone

A 27-year-old male officer, who was previously healthy and lived in South China, was presented to a local clinic in early November after skin itching, emesis, generalized myalgia and slight weakness in all limbs (Time line in Fig. 1). He denied any recent trauma, homosexual behavior, exposure to toxins, illicit substances, sick people, arthropods, or rodents. Additionally, he denied diet habit or history of eating fresh locally produced vegetables, snails, slugs, crabs and lizards. He had no known sick contacts but consumed a worm which accidentally entered the box with take-away smoked meat and was eaten by mistake one day before the onset. Given a suspicion of allergy and mild rhabdomyolysis, he was treated with anti-allergic agents and rehydration.

**Fig. 2:**
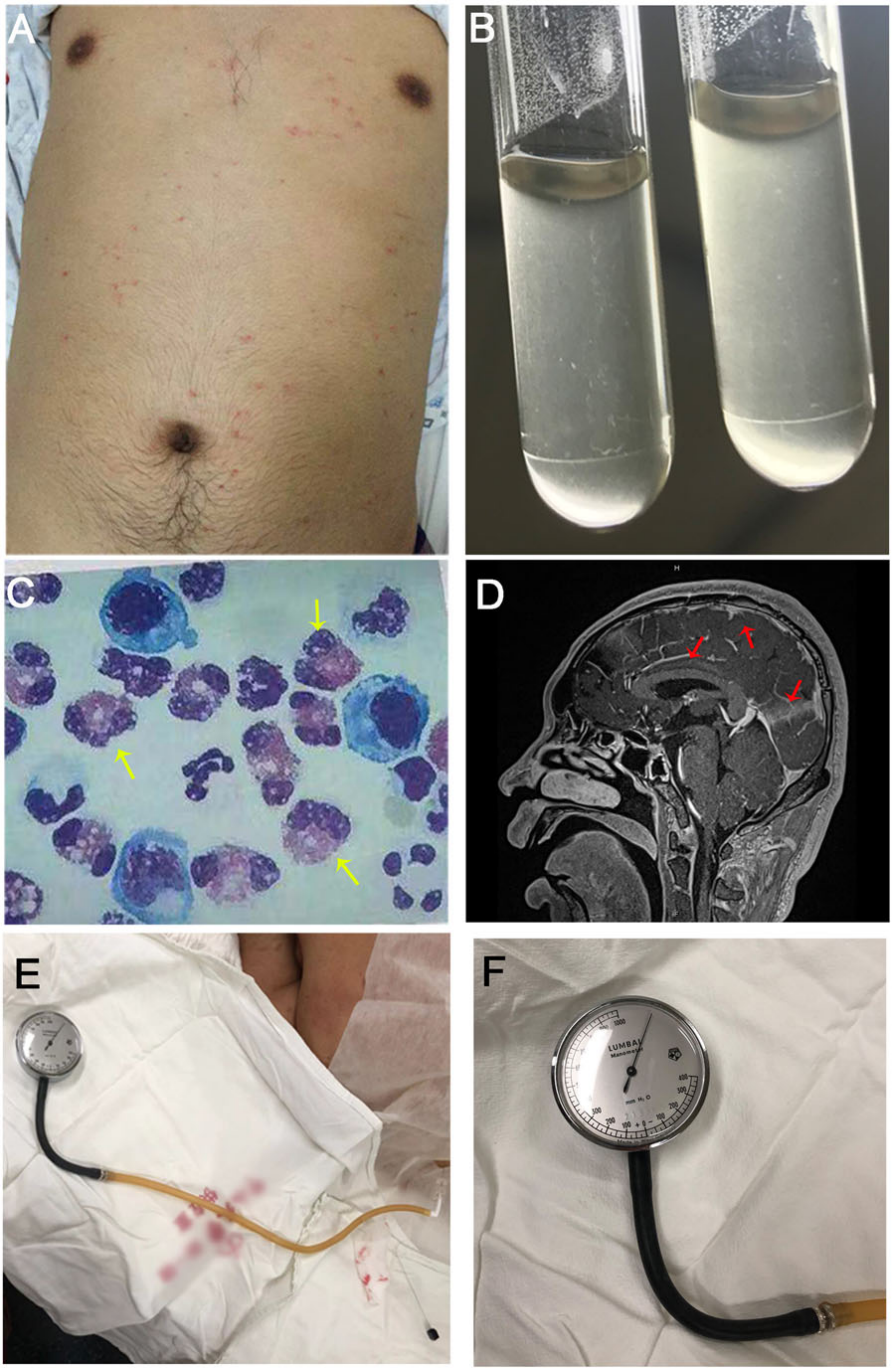
Clinical features of the patient with severe CNS angiostrongyliasis. **a** Generalized rashes throughout the trunk; **b** Coconut-like CSF; **c** Increased CSF eosinophils (yellow arrow); **d** The enhancement of meninges on T1WI (red arrows); **e** & **f** The extremely high CSF opening pressure over 1100mmH2O

Five days after onset (DAI 6), the patient manifested progressive pain and weakness of all limbs but alleviated itching. Upon admission to a local hospital, he presented muscle strength of Grade 3 for all limbs in physical examination. Transient rashes on his trunk were observed on DAI 8 (Fig. 2a). The CSF opening pressure was mildly elevated (230 mm H2O) by lumbar puncture (LP) on DAI 11, with the white blood cell (WBC) 164 × 10^6/L (71% monocytes and 29% multinucleated cells), protein 2380 mg/L and glucose 3.75 mg/L. CSF/blood glucose ratio was 71.1%. Gram stain was negative for bacteria. Fungal stain was negative for Cryptococcus, and no worms were seen in the CSF. Microbiological tests and computed tomography (CT) of the brain were unrevealing. He was then treated as Guillain-Barré syndrome with a high dose of intravenous methylprednisolone (40 mg/d) and immunoglobulin (0.4 g/kg/d) from DAI 11 to DAI 15, showing a significant but temporary improvement.

On DAI 15, the patient’s condition suddenly deteriorated with fever, generalized flaccid paralysis, gatism, and mental aberrations, including delusion and raves. The second LP was performed immediately to find CSF opening pressure remaining at 230mmH2O. CSF analysis revealed WBC 278 × 10^6/L (85% monocytes and 15% multinucleated cells). The CSF protein was 620 mg/L and glucose was 3.2 mg/L with CSF/blood glucose ratio of 58.6%. Cytology of CSF was not recorded. The mNGS for pathogen detection from the serum was carried out on DAI 16 and reported negative on DAI 18 (See Additional file 1). The serum enzyme-linked immunosorbent assay (ELISA) for parasites also negative. CT of the abdomen showed the peritoneum was focally thickened, indicating the possibility of peritonitis. Ceftriaxone and linezolid were thus prescribed immediately as the anti-infection therapy.

On DAI 17, the patient was transferred to our intensive care unit (ICU) for further treatment. He was sleepy but alert, complained of severe weakness, mild headache and stomachache. Neurological examination was remarkable for severe quadriparesis (muscle strength of Grade 1) and positive meningeal irritation signs. Extensive microbiological tests of the serum were negative on admission. He was intubated on DAI 20. The LP was performed again on DAI 20 and revealed raised CSF opening pressure of 520mmH2O and mNGS with the CSF sample was carried out for the first time. Of note, the CSF showed the coconut-like appearance (Fig. 2b), and the analysis of CSF disclosed eosinophils proportion was 67% (Fig. 2c), with the leucocytes increased to 750 × 10^6/L. On the same day, the patient fell into a persistent coma with the Bispectral index fluctuating between 50 to70. Given the consideration of worsening infection, ceftriaxone was replaced by meropenem. Ophthalmological consult recorded significant papilloedema but no evidence of parasitic invasion. As a result of the multidisciplinary consultation on DAI 21, dexamethasone (10 mg per day) and albendazole (400 mg per day) were added to the patient’s treatment regimen, with the consideration that parasitic infection was a potential etiology.

**Fig. 3:**
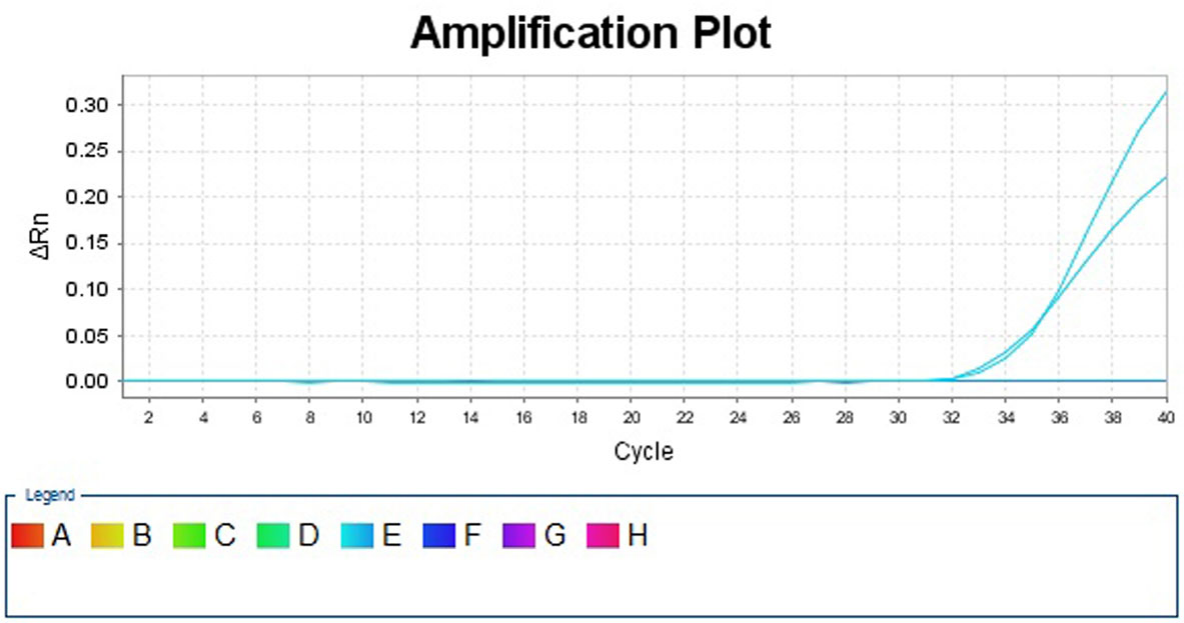
*A. cantonensis* identified by qPCR with the CSF on DAI 21

**Fig. 4:**
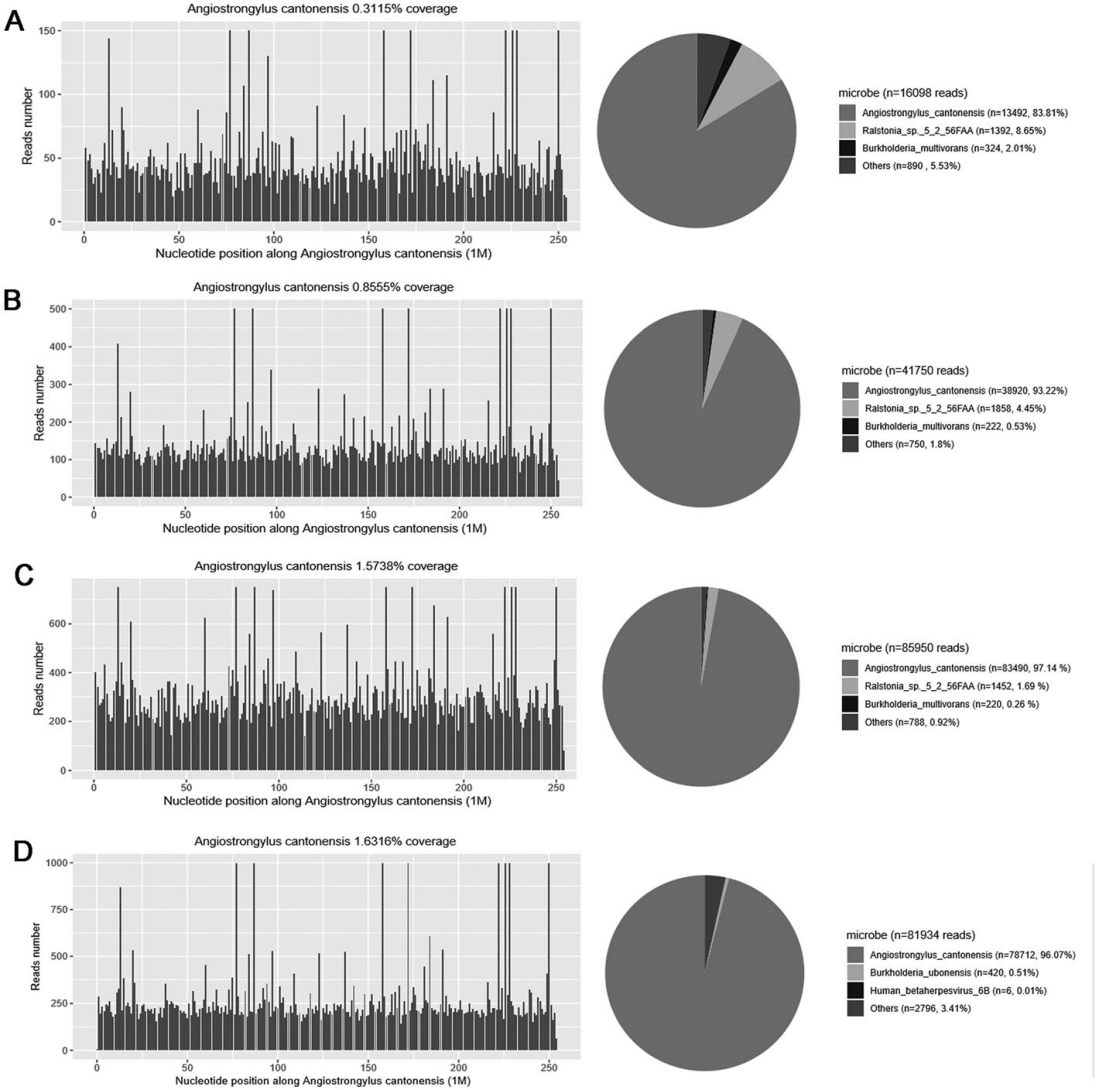
The cover charts of CSF mNGS for diagnosis. **a** Results of the mNGS for diagnosis on DAI 22. The DNA SMRN of *A. cantonensis* counted 83.81% among all microbes detected; **b** Results of mNGS after half-dose albendazole on DAI 25. The DNA SMRN of *A. cantonensis* counted 93.22% among all microbes detected; **c** Results of the mNGS after conventional dose of albendazole combined with methylprednisolone pulse on DAI 31. The DNA SMRN of *A. cantonensis* counted 97.14% among all microbes detected; **d** Results of the mNGS after intrathecal dexamethasone on DAI 36. The DNA SMRN of *A. cantonensis* counted 96.07% among all microbes detected

On DAI 22, *A. cantonensis* was reported for the first time by mNGS from the CSF with the DNA strictly map reads number (SMRN) of 13,492. The result was verified by quantitative polymerase chain reaction (qPCR) later with the same CSF sample for mNGS (Fig. 3) (Experimental details of mNGS and qPCR in Additional file 2). With the permit of his wife, the patient was treated by standard regimen of albendazole (400 mg twice a day) and high-dose intravenous methylprednisolone (500 mg per day) since DAI 23 according to the result of mNGS. On the other hand, mNGS was then carried out periodically to assess the condition during treatment (Fig. 4). Moreover, magnetic resonance imaging (MRI) on DAI 24 demonstrated diffuse enhancement of the meninges and cervical leptomeninges, with multiple new lacunar infarctions, scattered tiny hemorrhages, and prominent Virchow-Robin spaces (Fig. 2d). *A. cantonensis* was not confirmed by the enzyme-linked immunosorbent assay (ELISA) until DAI 31.

**Fig. 5:**
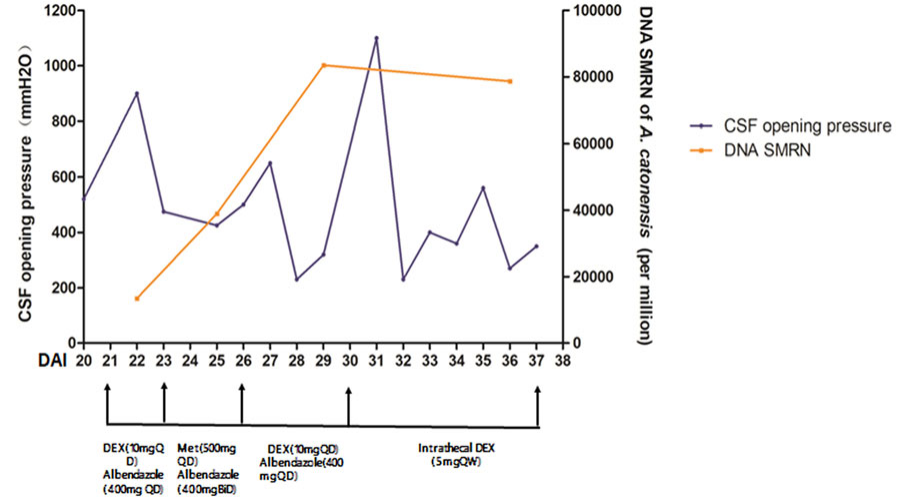
Change of the CSF opening pressure and SMRN of *A.cantonensis* along with treatment. DAI: day after ingestion; Dex: dexamethasone; Met: Methylprednisolone

Unfortunately, methylprednisolone was suspended on DAI 26 due to severe bleeding of pre-existing hemorrhoids and then the intrathecal dexamethasone was tried on DAI 31 because the miserable fact that the CSF opening pressure increased to more than 1100 mm H2O (Fig. 2e & f) and the patient was in deep coma with the Bispectral index falling to 20–30. The high CSF opening pressure then showed a drastic drop after intrathecal dexamethasone and eventually stabilized (Fig. 5). On the other hand, the Bispectral index recovered to 30 to 40 and the patient’s vital signs became relatively stable with less intravenous norepinephrine. The DNA SMRN of mNGS during treatment disclosed a sharp increase since anthelmintic therapy and a trend of slight decline after intrathecal dexamethasone (Fig. 5). However, the patient never regained his consciousness. He eventually died on DAI 38, right after the family abandoned therapy and discharged voluntarily, 16 days after the NGS diagnosis and 37 days after onset of symptoms.

## Discussion and conclusions

Although most human angiostrongyliasis manifests as eosinophilic meningitis and is of good prognosis with in-time treatment with corticosteroids and/or anthelmintics, CNS angiostrongyliasis with delayed treatment or untreated may developed into fatal meningoencephalitis. Given the fact that there is no valid treatment of severe CNS angiostrongyliasis, the early diagnosis and intervention accordingly becomes extremely essential for patients with angiostrongyliasis to survive. Although the discovery of larvae from the CSF or eye chamber is the gold standard for the diagnosis of CNS angiostrongyliasis, these findings are quite rare even in the severe cases [2]. Currently, angiostrongyliasis is usually considered based on the history of mollusk consumption, clinical features, eosinophilic pleocytosis in the CSF, and identification of positive antibody [10]. The increase of CSF eosinophils often follows an incongruent timeline to symptoms onset. The peak concentration of eosinophils in both CSF and peripheral serum has been reported at DAI 25–35 [8]. Immunological methods and PCR, such as ELISA, dot immunogold filtration assay (DIGFA) and PCR based on the 18S rRNA, were successfully applied for the identification of *A. cantonensis* [11–13]. However, positive results of ELISA or DIGFA can hardly be obtained in the acute phase of the disease. The animal experiments reveal the peak antibody response occurs 4 weeks after infection [14, 15]. Reverse transcription-PCR (rt-PCR) is of a specificity of 100% and advancing sensitivity but not general availability [16]. Moreover, the median time from onset to positive PCR result is 21 days, which is beyond the advised time window for anthelmintics [7, 16]. Last but not least, the effectiveness of all these target-dependent tests is compromised due to their dependence on the clinicians’ awareness of specific pathogens. Due to facilitated transportation and increasing interaction between districts throughout the world, more and more infectious diseases may break through their original endemic areas and spread all over the world, beyond the expectation of clinicians. Conventional microbiological testing is considered insufficient to detect all neuroinvasive pathogens [17]. In recent years, mNGS has become generally available for clinicians and it is suggested as a very powerful tool to detect the nucleic acids of all organisms in multiple biological materials. The target-independent identification of pathogens greatly facilitates the diagnosis of intracranial infectious diseases because it could provide important clues of all possible pathogenic microbes and assist clinicians in terms of diagnosis and treatment [9, 17, 18]. The new research on mNGS in severe CNS infectious diseases reported that mNGS contributed in diagnosing cases which were not identified with clinical testing and guiding treatment [17]. A prospective study reported that the sensitivity of mNGS in identifying bacteria, fungi, virus and tuberculosis in CSF was 73.3, 80, 76 and 66.7%, respectively, whereas the specificity of mNGS in diagnosing bacteria, fungi and tuberculosis in CSF was 95.9, 79.3 and 96.4%, respectively [19]. Comprehensively, mNGS was reported to achieve 73–92% sensitivity and 96–99% specificity compared to the routine laboratory testing, with 81% positive percent agreement and 99% negative percent agreement in terms of detecting pathogens in CSF [20]. In one word, either the positive or the negative results of mNGS could provide profound assistance to clinicians to make decisions.

In the current case, *A. cantonensis* was first detected by mNGS from the cerebrospinal fluid on DAI 20. After the identification of *A. cantonensis* by mNGS in the CSF, other definitive testing, including specific antibody, Charcot-Leyden crystals, reported positive. It is suggested that the mNGS showed high sensitivity in detecting *A. cantonensis* compared with other concurrent laboratory testing, and the target-independent identification of pathogens could aid clinicians to achieve definitive diagnosis even the clinician was unaware of the etiology. Therefore, mNGS did facilitate the identification of pathogens in the case. On the other hand, although *A. cantonensis* was reported to be detected with mNGS in a previous case, its existence was not confirmed with PCR [18]. In our case, the discovery of *A. cantonensis* by mNGS was verified by PCR, which provided solid evidence to support the workability of mNGS in identifying *A. cantonensis.*

Interestingly, the mNGS with serum sample on DAI 16 was negative in this case. A previous study has proved that mNGS with serum sample along could be false negative in meningitis and encephalitis [17]. As known, the larvae would molt and migrate from the intestines, passing through the liver, lungs, and finally reach the central nervous system within two weeks after ingestion, causing various but non-specific symptoms including enteritis, cough, developed fever, eosinophilic meningitis [2]. Therefore, the negative serologic identification of *A. cantonensis* by mNGS can be explained by the absence of nucleic acid of the causative pathogen in the mid-phase serum, where all larvae had left and migrated into the CNS. It is suggested that the effectiveness of samples may have a time window which is based on the life cycle of parasites. For detection of *A. cantonensis*, we suggest the CSF samples must be included for mNGS and the serum samples should not be tested alone once beyond the time window of two weeks.

*A. cantonensis* does not multiply but migrates, grows and dies in the CNS of humans [7]. The natural death of larvae in the CNS usually occurs approximately 1–2 months after migration into the CNS [21]. In our case, we recorded the change of DNA SMRN of *A. cantonensis* by mNGS since the definitive diagnosis in order to deepen the understanding of angiostrongyliasis and its treatment (Fig. 5). The increase of SMRN after anthelmintic treatment might suggest the growing segments of perished larvae, while the decline of SMRN was consistent with the improved vital signs after intrathecal dexamethasone. Besides, as Fig. 5 showed, the fluctuation of DNA SMRN of *A. cantonensis* varied in general accordance with the CSF opening pressure. The relationship between fluctuation of DNA SMRN and clinical outcome is worth further exploring.

The case of *A. cantonensis* infection highlights the benefit of mNGS as a target-free identification in disclosing the rare CNS angiostrongyliasis in the unusual season, while solid evidence from routine clinical testing was absent. Given the expanding endemic area of *A. cantonensis* due to the increasing interaction between districts and global warming, clinicians should be aware of the possibility of angiostrongyliasis and choose appropriate samples for mNGS according to the life cycle of *A. cantonensis*. Whether the DNA SMRN, which varied with CSF opening pressure and clinical manifestations, could be applied to monitor disease progression and treatment effectiveness is worth further observation.

## Supplementary information

**Additional file 1 **The cover chart of serum mNGS. Additional file 1 provided the cover chart of serum mNGS on DAI 16 which was negative in revealing *A. cantonensis.*

**Additional file 2.** Procedure of mNGS and PCR. Additional file 2 provided detailed procedure of mNGS and PCR.

## Data Availability

The datasets used and/or analyzed during the current study are available from the corresponding author on reasonable request.

## Abbreviations

CNS: Central nervous system
mNGS: Metagenomic next-generation sequencing
CSF: Cerebrospinal fluid
DAI: Day after ingestion
LP: Lumbar puncture
WBC: White blood cell
CT: Computed tomography
ICU: Intensive care unit
SMRN: Strictly map reads number
qPCR: Quantitative polymerase chain reaction
MRI: Magnetic resonance imaging
ELISA: Enzyme-linked immunosorbent assay
DIGFA: Dot immunogold filtration assay
rt-PCR: Reverse transcription-polymerase chain reaction
