# Supplementary material for: The metagenomic next-generation sequencing in diagnosing central nervous system angiostrongyliasis: a case report
Source: BMC Infect Dis. 2020 Sep 21;20:691. doi: 10.1186/s12879-020-05410-y (PMC7507257; doi:10.1186/s12879-020-05410-y)
Supplement: Supplementary file 1 — Additional file 1 The cover chart of serum mNGS. Additional file 1 provided the cover chart of serum mNGS on DAI 16 which was negative in revealing A. cantonensis. [file 12879_2020_5410_MOESM1_ESM.docx]

**Additional File 1**

**
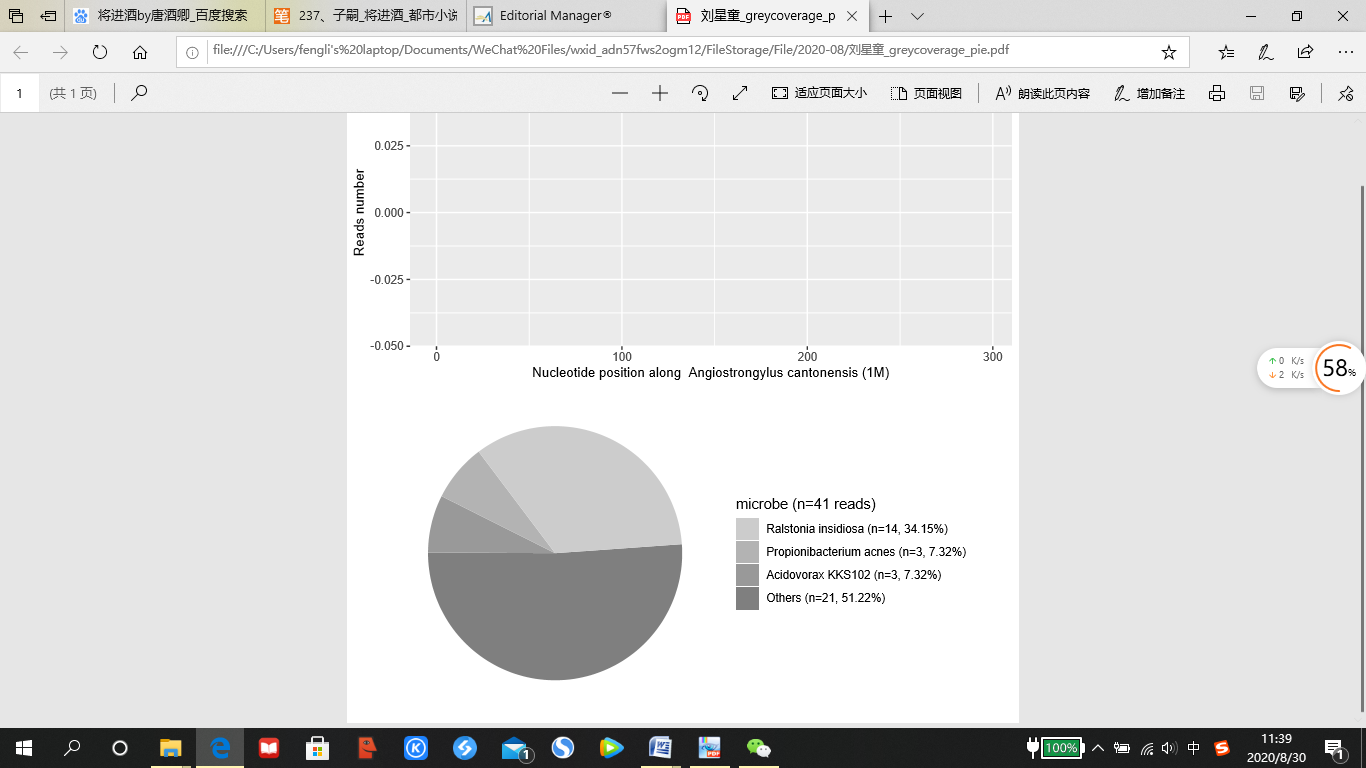
**

**Figure: The cover chart of serum mNGS.** Result of the serum mNGS for diagnosis on DAI 16. The DNA SMRN of A. cantonensis was not found at all.
