# Supplementary material for: The metagenomic next-generation sequencing in diagnosing central nervous system angiostrongyliasis: a case report
Source: BMC Infect Dis. 2020 Sep 21;20:691. doi: 10.1186/s12879-020-05410-y (PMC7507257; doi:10.1186/s12879-020-05410-y)
Supplement: Supplementary file 2 — Additional file 2. Procedure of mNGS and PCR. Additional file 2 provided detailed procedure of mNGS and PCR. [file 12879_2020_5410_MOESM2_ESM.doc]

**Additional file 2**

**Procedure of mNGS and PCR**

**1. Methods and procedure of mNGS**

Cerebrospinal fluid was collected by lumbar puncture and stored in a sterile container. Cerebrospinal fluid specimen was preserved through dry ice and transported to the laboratory.

The DNA of CSF specimen was extracted by TIANGEN DP316 kit, together with a negative control (RNase-free water) and a positive control. Next, the DNA libraries were prepared using NEBNext Ultra II DNA library Prep Kit. The DNA libraries concentration was measured by Qubit. Then the sequencing was done by illumina miniseq system with 150 cycles Reagent Kit.

After obtaining the sequencing data, high-quality data were generated after filtering out adapter, low-quality, low-complexity, and shorter reads. Next, remove human reads by mapping reads to human reference genome. The remaining data were aligned to the microbial genome database using Burrows-Wheeler Alignment. Finally get the microbial compositions of the samples. The microbial genome database contains more than 20,000 microorganisms, including bacteria, viruses, fungi and parasites. The pathogen detection pipeline described above was compared with SURPI [1], and there is a high consistency of results.

The sampling, storage and transportation process strictly follows aseptic procedures to ensure that qualified CSF specimens are collected. Both Nucleic acid extraction and library preparation were conducted in parallel with quality control samples. To eliminate background interference, a minimum threshold of 10 RPM-r (RPM defined as Reads per million, RPM-r defined as RPMsample/RPMNTC) was designated for reporting the detection of a microorganism as “detected” [2].

All detection procedures for pathogenic microorganisms are completed within 48 hours after receiving specimen in the laboratory, ensuring timely guidance for clinical treatment.

# 2. Limitations

The sampling process may be contaminated by skin, environment and sampling operation. This sampling process requires strict aseptic procedures. We collected the middle part of the cerebrospinal fluid. The Specimens need to be kept at low temperature to prevent nucleic acid degradation. Once the specimens were collected, we transported them to the lab via dry ice. The specimens were kept in the -80 degree refrigerator until the beginning of the experiment. The experimental results will be affected by the efficiency of nucleic acid extraction and the quality of library preparation. We selected the widely used nucleic acid extraction and library preparation kit. Sequencing can also affect data quality. We chose Illumina sequencing platform, which is known for its high quality. And we conducted strict quality control on sequencing data through data analysis.

The data analysis method will affect the test results. The main steps of data analysis include data filtering, human sequences removal and sequence alignment. If the data filtering conditions are too strict, many meaningful sequences may be removed. However, if the data filtering conditions are too loose, many low-quality sequences will be left to affect the results. We selected trimmomatic, the most widely used software, to filter the sequencing data. Clinical samples contain a large number of human source sequences, and the presence of these sequences will affect the efficiency and accuracy of microbial alignment. So the human sequences were usually removed first. We used SNAP software to do this. The sensitivity and specificity of sequence alignment software can also have a significant impact on the results. We chose BWA software, which is generally considered to be highly accurate. All software is inevitably biased. At each step, we chose the most widely used software that worked well. And we compared the results of this whole process with SURPI software. The results of the two pipelines are highly consistent.

Our database contains most of the microbial genomes that have been sequenced. Our goal is to cover a more comprehensive range of microbes. But we only use high-quality genomes, like completed genomes. This prevents the introduction of many faulty or problematic genomes. The limitation of the reference database is that it can only detect microorganisms that exist in the reference genome database. If the microorganism is not sequenced or not included in the database, it cannot be detected, and false negatives occur.

**3. Methods and procedure of qPCR**

qPCR was performed for verifying the result acquired from mNGS. The DNA of CSF specimen collected on the 25^th^ and 31^st^ days post assumed was extracted by TIANGEN DP316 kit, together with a negative control (RNase-free water) and a positive control. Next, a segment of the 232bp *A. cantonens*is gene was amplified with the following primers: forward primer 5’-CTCGGCTTAATCTTTGCGAC-3’, reverse primer 5’-CTCGGCTTAATCTTTGCGAC-3’. PCR amplification was in a 20μl volume with 10μl KAPA SYBR FAST qPCR Master Mix (2X) Universal, 0.4μl ROX Low Reference Dye (50X), 0.4μl forward primer and 0.4μl reverse primer, and 8.8μl template DNA. TB buffer was as a negative control. The amplification profile was initial denaturation at 95℃ for 3 minutes, a 40 cycles at 95℃ for 15s, 58℃ for 45s. A melt curve followed at the end.

**Refenrence**

[1] Naccache, Samia N., et al. "A cloud-compatible bioinformatics pipeline for ultrarapid pathogen identification from next-generation sequencing of clinical samples." Genome Research 24.7 (2014): 1180-1192.

[2] Miller, Steve, et al. "Laboratory validation of a clinical metagenomic sequencing assay for pathogen detection in cerebrospinal fluid." Genome Research 29.5 (2019): 831-842.

[3] Silva, Ana Cristina Arámburu da, Graeff-Teixeira C, Zaha A. Diagnosis of abdominal angiostrongyliasis by PCR from sera of patients[J]. Revista do Instituto de Medicina Tropical de São Paulo, 2003, 45(5):295-297.
